# Subdural haematoma in pregnancy-induced idiopathic thrombocytopenia: Conservative management

**DOI:** 10.4103/0019-5049.71036

**Published:** 2010

**Authors:** Maitree Pandey, Namita Saraswat, Homay Vajifdar, Lalita Chaudhary

**Affiliations:** Department of Anaesthesia, Lady Hardinge Medical College, New Delhi, India

**Keywords:** Conservative management, idiopathic thromocytopenic purpura, pregnancy, subdural haematoma

## Abstract

Conservative management of subdural haematoma with antioedema measures in second gravida with idiopathic thrombocytopenic purpura (ITP) resulted in resolution of haematoma. We present a case of second gravida with ITP who developed subdural haematoma following normal vaginal delivery. She was put on mechanical ventilation and managed conservatively with platelet transfusion, Mannitol 1g/kg, Dexamethasone 1mg/kg and Glycerol 10ml TDS. She regained consciousness and was extubated after 48 hrs. Repeat CT after 10 days showed no mass effect with resolving haematoma which resolved completely after 15 days. Trial of conservative management is safe in pregnant patient with ITP who develops subdural haematoma.

## INTRODUCTION

Gestational thrombocytopenia is the most common cause of thrombocytopenia, followed by idiopathic thrombocytopenic purpura (ITP). In gestational thrombocytopenia, the platelet counts are typically >70,000/ml, with about two-thirds being 130,000–150,000/ml. We have discussed how to manage Idiopathic thrombocytopenic purpura conservatively in pregnancy as surgical management is not possible due to low platelet count and if at all done it is associated with high morbidity and mortality.

## CASE REPORT

A 25-year-old, full term, second gravida was admitted in second stage of labour with severe anaemia (Hb: 5 g/dl) and thrombocytopenia. She had no previous antenatal reports. She was haemodynamically stable until the normal vaginal delivery, when she lost 1 l of blood. Two hours later, she developed respiratory distress, haemodynamic instability, drowsiness and became unconscious. It was attributed to blood loss and she was resuscitated with 1 l of colloids and 2 units of whole blood, but she remained unresponsive, was intubated and put on ventilator for elective ventilation (SIMV mode). She also developed bleeding from nose and gums. Her platelet count had dropped to 28,000/mm^3^, but prothrombin time and INR (International normalized ratio) were normal. Platelet antigen typing showed raised platelet-associated immunoglobulins (6.0, reference range 0–4) suggesting idiopathic thrombocytopenic purpura. An urgent computed tomography (CT) scan of the brain showed subdural haematoma (Lt temporal) with mass effect. Six units of platelet concentrate were transfused. The subdural haematoma was managed conservatively in view of severe symptomatic thrombocytopenia and the patient was managed with Mannitol 1 g/kg, Dexamethasone 10 mg/kg and Glycerol. She regained consciousness and was extubated after 48 hours. Repeat CT after 10 days showed no mass effect with resolving haematoma which resolved completely after 15 days.

## DISCUSSION

Gestational thrombocytopenia is the most common cause of thrombocytopenia, followed by ITP. In gestational thrombocytopenia, the platelet counts are typically >70,000/ml, with about two- thirds being 130,000–150,000/ml. Thrombocytopenia in pregnant individuals may result from the effects of several diverse processes, which may be either physiological or pathological [Table 1]. It has been estimated that ITP affects 1–2 in every 10,000 pregnancies.[1,2] ITP is defined as isolated thrombocytopenia (low platelet count with otherwise normal results on complete blood count and peripheral blood smear) in a patient with no clinically apparent associated conditions or factors that can cause thrombocytopenia. An abnormal blood count or peripheral blood smear due to a coexisting nonimmune condition (such as iron deficiency or thalassemia minor) does not, in itself, exclude the diagnosis of ITP. The diagnosis of ITP is based principally on the exclusion of other causes of thrombocytopenia using the history, physical examination, blood count, peripheral blood film, autoimmune profile and other investigations.[3]

**Table 1:** Causes of thrombocytopenia in pregnancy[3]

Isolated thrombocytopenia Gestational Immune (ITP) Drug-induced Type IIb von Willebrand disease Congenital Thrombocytopenia associated with systemic disorders Pregnancy-specific Pre-eclampsia HELLP (haemolysis, elevated liver function tests, low platelets syndrome) Acute fatty liver Not pregnancy specific Thrombotic microangiopathies Thrombotic thrombocytopenic purpura Haemolytic uraemic syndrome Systemic lupus erythematosus Antiphospholipid antibodies Disseminated intravascular coagulation Viral infection [human immunodeficiency virus (HIV), Epstein-Barr virus (EBV), cytomegalovirus (CMV)] Bone marrow dysfunction (primary or secondary) Nutritional deficiency Hypersplenism

Our patient’s platelet count was 28,000/mm^3^ and her platelet-associated immunoglobulins were high, at 6.0 (reference range 0–4), favouring the diagnosis of ITP.

Maternal haemorrhage at the time of birth is a risk in women with ITP, particularly, if the platelet count decreases to less than 20,000/μl. Intracerebral haematoma or subarachnoid haemorrhage is more frequently reported in these patients. Subdural haematoma usually occurs as an extension of a parenchymal bleed. Isolated subdural haematoma is an extremely rare entity in ITP. So far, only very few cases have been reported in the literature.[4,5] The basic pathology in thrombocytopenia is proposed to be capillary leak and this may become confluent in severe cases, leading to frank intracerebral haematoma. This intracerebral source of bleeding probably explains the rarity of subdural haematoma in ITP.[6] Because of the absence of associated parenchymal injury, the outcome may be better in non-traumatic acute subdural haematoma (ASDH). For the same size of subdural haematoma, chances of complications like brain oedema and herniation may be less in non-traumatic ASDH. Trial of conservative management may be safe in selected situations. More and more cases are being reported of patients being managed conservatively under these circumstances.[4,7] The recovery of our patients with conservative management further supports these facts.
